# Comparison of the antisclerotic effect of hydroalcoholic extracts of *Ocimum basilicum *and *Otostegia persica* with quinacrine by inhibition of phospholipase A_2_ in male Wistar rats

**DOI:** 10.22038/AJP.2021.19075

**Published:** 2022

**Authors:** Fatemeh Ghanbarpour Rahdari, Kahin Shahanipour, Ramesh Monajemi, Mohammad Adibnejad

**Affiliations:** 1 *Department of Biochemistry, Falavarjan Branch, Islamic Azad University, Isfahan, Iran*; 2 *Department of Biology, Falavarjan Branch, Islamic Azad University, Isfahan, Iran*

**Keywords:** Hypercholesterolemia Antisclerotic, Otostegia persica, Ocimum basilicum Phospholipase A2

## Abstract

**Objective:**

Hypercholesterolemia is now considered a major risk

factor for development of atherosclerosis. The phospholipase A2 superfamily of enzymes has causal involvement in atherosclerosis. Atherosclerosis is one of the main causes of mortality in developed countries and in some developing countries such as Iran. The present study was designed to investigate the antihypercholesterolemic and antiatherogenic potentiality of ethanolic extracts of *Ocimum basilicum* (*O. basilicum*) and *Otostegia persica* (*O. persica*) in high-fat diet-induced hypercholesterolemic rats.

**Materials and Methods:**

In this study, 35 male rats were randomly divided into 1 normal diet and 4 high-fat diet groups. After two months of high-fat diet, measurement of cholesterol and LDL showed a significant difference between the groups. The 5 groups were as follows: Healthy rats receiving physiological serum, hypercholesterolemic rats without any treatment, hypercholesterolemic rats receiving quinacrine (30 mg/kg), hypercholesterolemic rats treated with extract of *O. persica* (300 mg/kg), and hypercholesterolemic rats treated with *O. basilicum* extract (300 mg/kg). Treatment was carried out for 40 days and finally, blood samples were collected and examined for cholesterol, triglyceride, high density lipoprotein, low density lipoprotein, C-reactive protein, phospholipase A_2_**,** and interleukin-6 levels.

**Results:**

Treatment of hypercholesterolemic rats with ethanolic extracts of *O. persica* and *O. basilicum* did not cause significant changes in cholesterol, triglyceride and LDL or HDL levels. They caused a significant decrease in the levels of inflammatory factors of IL-6, PLA2 and CRP (p <0.05).

**Conclusion:**

Ethanolic extracts of *O. persica* and *O. basilicum* have antisclerotic effects by reducing the inflammatory factors and PLA2 activity.

## Introduction

Nowadays, hypercholesterolemia is a major risk factor for development of atherosclerosis. Today's lifestyle is associated with high-cholesterol diet and lack of physical activity. It leads to hypercholesterolemia and changes in serum lipids which increase the risk of cardiovascular disease (Mile et al., 2008 ▶; Ghannadi et al., 2015 ▶). The pathogenesis of atherosclerosis is complex and there is evidence suggesting that oxidation of lipids and low density lipoprotein (LDL) is one of the important events involved in the formation of atherosclerotic plaques. The use of oxidants in food produces oxidized LDL which causes the development and progression of atherosclerosis. When LDL is oxidized, its affinity for the receptor decreases and accumulation of oxidized LDL in macrophages leads to formation of foam cells and subsequently, atherosclerosis (Ahmadvand et al., 2011 ▶). Examination of lipid profile is of special importance in the diagnosis and follow up of atherosclerosis. Some people who are genetically predisposed to atherosclerosis, or people with risk factors such as high level of cholesterol, triglycerides, and other harmful fats, gradually develop endothelial damage and deposition of these compounds under the arterial endothelium which results in atherosclerosis (Azizi et al., 2004 ▶).

According to Russell Ross theory, atherosclerotic lesions mainly consist of macrophages and T lymphocytes. These cells have very specific molecular and cellular responses that can be explained by the inflammatory nature of atherosclerosis. A set of acute phase proteins, cytokines, and soluble cellular binding molecules are involved in causing these lesions (Vakili et al., 2013 ▶; Tabas, 2017 ▶). Increased concentrations of cholesterol and triglycerides and decreased HDL in rabbit receiving high-fat diet by affecting the metabolism of arachidonic acid and stimulating leukocytes, increase the production of free radicals (Lee et al., 2003 ▶).

Increasing levels of free radicals reduce the synthesis of nitric oxide in endothelial cells. Reduction of nitric oxide damages the relaxation associated with smooth muscle endothelium and makes the vessel prone to plaque formation (Asgary et al., 2006 ▶).

The observational studies have shown that phospholipase A2 (PLA2) concentration and activity predict cardiovascular events. The sn-2 ester bond of phospholipid substrate is hydrolyzed by PLA2. Released free fatty acid and lysophospholipid have direct proinflammatory effects. Eicosanoids are a side product from released arachidonic. In addition to its indirect effects on fostering cholesterol crystal formation, PLA2 increases oxidative stress through generation of arachidonic acid, lysophospholipids, and nonesterified fatty acids. These bioactive lipids, including the arachidonic acid, which produce prostaglandins, thromboxanes, and leukotrienes, work collectively with oxidized LDL to recruit inflammatory cells to atherosclerotic lesions and activate inflammatory pathways in multiple cells in the vessel wall. Secretory PLA2 activity can be proatherogenic in the circulation, and at sites of atherosclerotic plaque development. Thus, in atherosclerotic plaques, these products can reach high local concentrations (Burke and Dennis, 2009 ▶).

Recently, selective inhibitors of PLA2 have been targeted as potential candidates to reduce atherosclerotic cardiovascular events (Rosenson and Hurt-Camejo, 2012 ▶).

Research has shown that some plants with properties such as controlling oxidation, regulating blood lipids and reducing inflammation, are able to inhibit the formation stage and even the progression of atherosclerosis through various mechanisms such as controlling the formation of fat veins (Which is the first step in the onset of atherosclerotic lesions).

In fact, the formation of fat streaks that begin after the invasion of macrophages and the transfer of lipoproteins, especially LDL cholesterol and LDL oxide particles into the inner space of the vessel wall, seems to be a controllable phenomenon (Heinecke et al., 2001 ▶). Medicinal plants are rich sources of natural antioxidants and are used in traditional medicine to control and treat many diseases (Behradmanesh et al., 2012 ▶).

The genus *Otostegia* of the family Lamiaceae includes 20 species, which are distributed in West Asia. *Otostegia persica* is native to Iran, Pakistan and Afghanistan (Hedayati et al., 2012 ▶). Extracts of the aerial parts of *O. persica* have anti-histamine, antispasmodic and anti-arthritic properties (Dourandishan et al., 2014 ▶).

*Ocimum basilicum* grows naturally in tropical and subtropical regions, especially in Asia, Africa, Central and South America (Labra et al., 2004 ▶). *O. basilicum* has several pharmacological effects such as anti-giardiasis, trypanocidal, anti-*Helicobacter pylori*, anti-viral, anti-oxidant and anti-hyperlipidemic properties (Niazi et al., 2017 ▶).

Considering the importance of lipid profile and inflammatory factors C- reactive protein (CRP) and interleukin-6 (IL-6) and increasing function of PLA2 in the process of inflammation and the use of chemical drugs to reduce these factors, we decided to examine the effect of the extracts of *O. persica* and *O. basilicum* on reducing the desired factors and demonstrate whether relying on these plants can reduce inflammation and the risk of developing atherosclerosis.

## Materials and Methods

*O. persica* was collected in December 2017 from pastures around Jam city located in Bushehr province and dried in the shade by drying method. *O. basilicum* was prepared from natural sources of Isfahan in dried form. The identification of the plants was done by the herbarium in department of Islamic Azad University, Falavarjan branch, Isfahan, Iran. First, the dried plants (*O. persica* and *O. basilicum *with 23332 and 114.001.001 herbarium code respectively) were crushed by a home mill and then the extraction was done by maceration and 99.8% ethanol. 

This study was performed on 35 male Wistar rats with a weight range of 180 to 220 g in the animal house and research laboratory of Falavarjan Azad University. Rats were obtained from the laboratory animal breeding center of Falavarjan Azad University. Rats were allowed to adjust to the pet's condition for one week before the start of the experiment. Rats were kept in an animal house with a light cycle of 12 hr of darkness, 12 hr of light and a temperature of 22°C with a standard diet during the research period to achieve a suitable weight. The drinking water of the animals was urban tap water throughout the experimental period. All ethical points regarding keeping and working with animals were observed in the laboratory. For inducing hypercholesterolemia in rats, a high-fat diet was used. The animals were divided into 5 groups of 7, including a group that received a normal diet and four groups that received a high-fat diet. The high-fat diet used consisted of 1.5 g of cholesterol powder and 3.5 g of fat, which was mixed with 95 g of rat food and given to rats for two months, after which intermediate sampling was performed. By observing ethical principles blood samples were taken from the corners of the eyes and cholesterol and LDL of these rats were measured to ensure that they were hypercholesterolemic and then the required amount of extract according to the average weight of rats and the treatment period was calculated. The hydroalcoholic extracts of *O. persica* and *O. basilicum* at concentrations of 300 mg/kg were given to rats by gavage for 40 days (Hedayati et al., 2012 ▶; Rasekh et al. 2011 ▶; Al Asmari et al., 2017 ▶).

The experimental groups in this study were: Group 1: control (healthy rats receiving physiological serum). Group 2: high-fat (rats receiving high-fat diet, but no extracts or drugs). Group 3: High-fat diet and quinacrine (30 mg/kg). Group 4: Recipients of high-fat diet and *O. persica* extract (300 mg/kg). Group 5: Recipients of high-fat diet and *O. basilicum* extract (300 mg/kg).

On the 40th day, animals were deeply anesthetized using a cocktail of 50 mg/kg ketamine and 4 mg/kg xylazine (ip), then, blood samples of each animal were collected through the portal vein of the heart. After centrifugation of blood samples at 2200 rpm for 15 min and preparation of serum, the level of total cholesterol, triglyceride, LDL, and HDL was measured using kits made by Pishtaz Teb Company by an autoanalyzer. The amount of IL-6 and CRP was measured using kits made by Hangzhou Eastbiopharm Company and by ELISA method and the activity of PLA2 enzyme was measured by a kit made by Bioassay Technology Laboratory Company by ELISA sandwich method.

This dissertation with the code IR.IAU.FALA.REC.1397,025 has been approved by the ethics committee of Islamic Azad University, Falavarjan branch.

**Statistical analysis**

The data are presented as mean±SD obtained from the experiment were statistically analyzed using SPSS software version 21. To compare the mean of each of the factors between control and high-fat groups, first the normality of the data was analyzed using the Kolmogorov-Smirnov test. If the data are normal, independent-Samples T Test or otherwise Mann-Whitney U nonparametric test was used.

For end-of-work, it means comparing the mean of each parameter in the studied groups (control, *O. persica* extract, *O. basilicum* extract and quinacrine), if the assumptions are established (normality and homogeneity of data variance), one-way analysis of variance test (One-Way ANOVA) or otherwise the non-parametric Kruskal-Wallis test was used. In addition, a significance level of less than 0.05 was considered for all analyses.

## Results

In this study, high-fat diet was given to four groups of rats for two months, after which intermediate sampling was performed and cholesterol and LDL levels were compared with the control to make sure that they are hypercholesterolemic.

**Figure 1:**
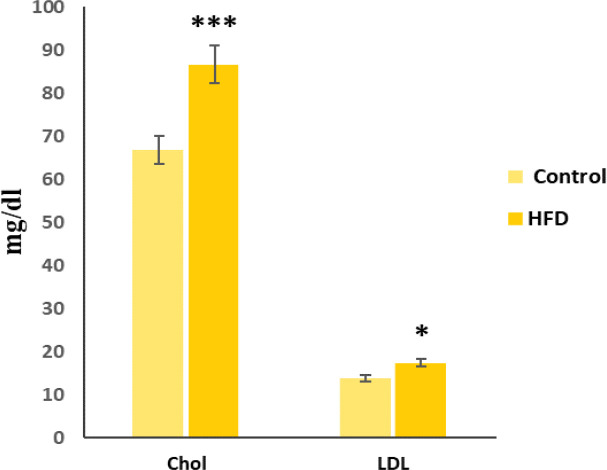
Mean±SD cholesterol and LDL level in the studied groups (n=7). ***p≤0.001 and *p<0.05) t-test (in comparison to the control group. Chol: cholesterol, LDL: low density lipoprotein, HFD: high-fat diet

According to Figure 1, the mean of intermediate cholesterol in the control group was significantly lower than the other group (t-test p ≤ 0.001).

The comparison of mean LDL intermediate sampling was performed using t-test of two groups. The mean of LDL in the control group was less than the other group and this difference was significant (p<0.05).

**Figure 2:**
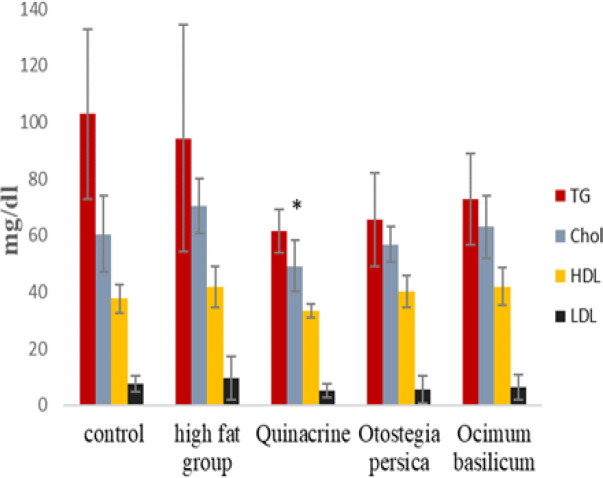
Mean±SD TG, Cho, HDL, and LDL levels in the studied groups. *p<0.05 (ANOVA) in comparison to the high-fat group (n=7).

According to Figure 2, no significant difference in mean triglyceride was observed between the groups. The mean triglyceride level was the highest in the control group but the difference with the high-fat group was not significant. Also, the difference between the mean of triglyceride in the high-fat group and the mean value of the other groups was not significant. Quinacrine lowered triglycerides to some extent, which was slightly more effective than the two plant extracts.

Comparison of mean cholesterol level among the study groups by analysis of variance test had a borderline result. The mean was the highest in the high-fat group and it was significantly different from the quinacrine group (p<0.05). The effect of *O. persica* and *O. basilicum* extract in lowering cholesterol was not significant compared to quinacrine, but the effect of quinacrine was greater than these two extracts.

The comparison of the mean HDL level among the groups had a borderline result (p-value=0.06 analysis of variance). With reference to post hoc tests, it was observed that the mean level of HDL in the *O. basilicum* extract and high-fat groups was the highest and the difference between the mean level of the high-fat and quinacrine group was significant. The effect of *O. persica* extract was almost equal to that of *O. basilicum*.

The comparison of the mean LDL level among the groups did not show a significant difference. The mean was highest in the high-fat group followed by the control group, but this difference is not significant. 

**Figure 3:**
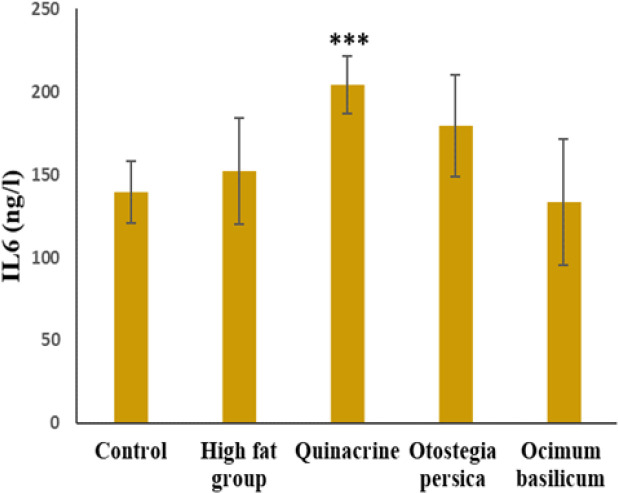
Mean±SD IL-6 level in the studied groups. ***p≤0.001 (ANOVA( in comparison to the high-fat group and *O. basilicum* group (n=7).

According to Figure 3, the mean level of IL-6 in the treatment groups was significantly different from the high-fat group (p<0.001). The mean of IL-6 was highest in the quinacrine group and it was significantly different from the high-fat group and *O. basilicum* extract. 

**Figure 4:**
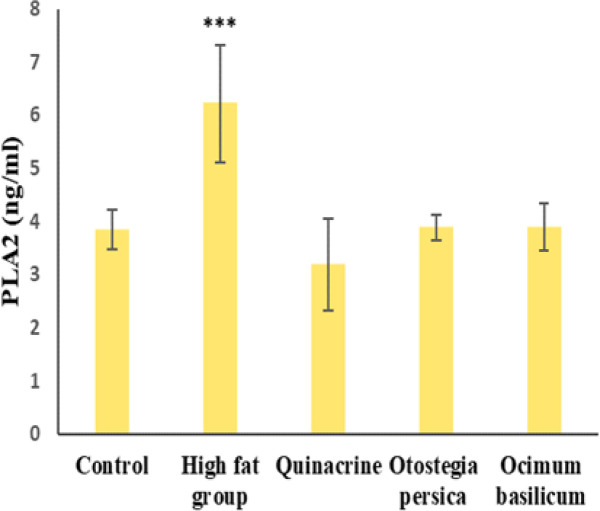
Mean±SD PLA2 level in the studied groups. ***p≤0.001 (ANOVA (in comparison to the control group (n=7).

**Figure 5:**
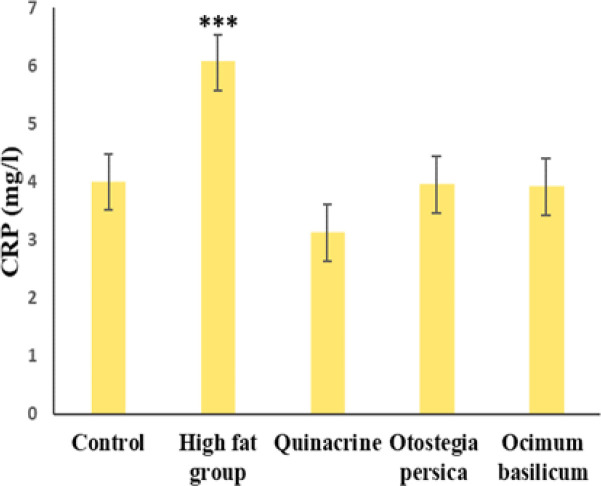
Mean±SD CRP level in the studied groups. ***p≤0.001 (ANOVA) in comparison to the control group (n=7)

According to Figure 4, the mean activity of PLA2 was significantly different among the studied groups (p<0.001). The mean was significantly different between the high-fat and control groups. The mean PLA2 level was the lowest in the quinacrine group and the highest in the high-fat group and they were significantly different from PLA2 levels in the other groups. The effect of *O. basilicum* and *O. persica* extract was almost the same in terms of PLA2 level, but somewhat less than the effect of quinacrine. According to Figure 5, comparing the mean CRP level among the groups showed a significant difference (p<0.001). The mean level of CRP was significantly different between the high-fat and control groups. The mean CRP level was the lowest in the quinacrine group and the highest in the high-fat group, and they were significantly different from CRP levels in the other groups. The changes in CRP levels under influence of O. basilicum and O. persica extracts are almost the same and less than the effect of quinacrine. 

## Discussion

The results of many studies show that despite many efforts to gain knowledge and practical strategies to reduce mortality from cardiovascular disease, this disease is one of the leading causes of death in many countries (Vasa et al., 2006 ▶). There is ample evidence that proinflammatory cytokines are more sensitive and accurate in predicting cardiovascular disease and play an important role in the pathogenesis of atherosclerosis (Pontiroli et al., 2004 ▶). CRP has been introduced as the most sensitive inflammatory indicator and a strong independent predictor of cardiovascular risk, which can be used to identify people prone to premature atherosclerosis (Michishita et al., 2008 ▶). CRP is synthesized in liver cells and is present in small amounts in the bloodstream; it is stimulated by inflammatory and infectious processes in the liver and enters the bloodstream (Jenabi et al., 2007 ▶).

IL-6 is a potent inducer of the acute phase response in the liver. Acute phase reaction is associated with increased fibrinogen levels, which is a strong risk factor for coronary artery disease. This cytokine plays an important role in the early stages of atherosclerosis (Yudkin et al., 2000 ▶).

PLA2 and lipoprotein-associated phospholipase A2 are related to atherogenesis and its complications. These two enzymes produce biologically active metabolites that are involved in various stages of the atherosclerosis process. Pathological studies in animals have shown that increased levels of these two enzymes are associated with increased coronary lesions and cardiovascular disease (Garcia et al., 2009 ▶).

Cell damage activates PLA2, which produces arachidonic acid and eventually leads to the production of eicosanoids. Eicosanoids are inflammatory mediators (Melo et al., 2012 ▶).

The results of this study showed that treatment of rats with hydroalcoholic extracts of *O. persica*, *O. basilicum* and quinacrine after 40 days of high-fat diet did not significantly lower triglyceride levels compared to high-fat. Hydroalcoholic extracts of *O. persica* and *O. basilicum* did not cause a significant reduction in cholesterol compared to the high-fat but quinacrine significantly reduced this factor. Hydroalcoholic extracts of *O. persica* and *O. basilicum* caused a significant increase in HDL compared to the high-fat, but the effect of *O. persica* extract and quinacrine was not significant compared to *O. basilicum* extract. 

Hydroalcoholic extracts of *O. persica *and* O. basilicum* and quinacrine caused a significant decrease in CRP and PLA2 compared to the high-fat group. Quinacrine did not significantly reduce the amount of IL-6 in comparison with the high-fat group, but the hydroalcoholic extracts of *O. persica* and *O. basilicum* caused a significant decrease in this factor compared to quinacrine. The reason for the decrease in triglycerides and cholesterol in the group receiving *O. persica* and *O. basilicum* extracts in rat fed with a high-fat diet can be attributed to the compounds in them especially quercetin and morin in *O. persica* and rosemarinic acid in *O. basilicum*. The reason for the decrease in IL-6 levels in rats receiving *O. basilicum* extract can be attributed to the phenolic compounds in it, especially eugenol, which have antioxidant properties. According to the results of the present experiment, it can be said that the reason for the decrease in PLA2 activity by *O. persica* is due to flavonoid compounds such as β-cytosterol or its eugenol compound, which can inhibit the enzyme cyclooxygenase-2. According to the results of the experiment, it can be said that *O. persica* and *O. basilicum* were able to reduce the inflammatory factor CRP due to their abundant levels of flavonoids and phenolics. A study showed that the use of quercetin and morin reduces triglycerides and cholesterol in the serum, liver and kidneys of rats and, as a result, their use is useful in improving atherosclerosis (Fabiane et al., 2001 ▶). Quercetin is able to inhibit the fatty acid enzyme synthase and cholesterol biosynthesis in liver cells (Yamamoto et al., 2006 ▶). It has been observed that the extracts of some plants reduce cholesterol and blood triglyceride levels by increasing the activity of hepatic α-7-cholesterol hydroxylase, increasing cholesterol excretion and reducing cellular cholesterol synthesis (Taghizadeh Afshari et al., 2007 ▶). 

High-density lipoprotein (HDL) has the activity of reversing cholesterol transfer from peripheral tissues and macrophages to hepatocytes, as well as antioxidant, anti-inflammatory, and antithrombotic activities. Concentration less than 40 mg/dl of HDL is thought of as lipid disorder and is considered in extensive epidemiological research as a strong predictor of cardiovascular disease (Ghasemnejad et al., 2013 ▶). Most of the antioxidant properties of HDL are related to the enzyme paraoxonase-1 (PON-1), which binds to HDL and travels with it in the blood. This enzyme is mostly produced in the liver and has anti-atherogenic properties that are achieved by biological hydrolysis of oxidized phospholipids (Zarei et al., 2017 ▶).

Examination of the anti-inflammatory effects of flavonoids, eugenol and β-cytosterols showed that these compounds are able to inhibit the enzyme cyclooxygenase (Mohammadi et al., 2017 ▶; Saeidnia et al., 2014 ▶; Asgary et al., 2015 ▶).

Eugenol is an unstable phenolic substance whose derivatives are used in medicine as antiseptics and local anesthetics. Eugenol has a wide range of antimicrobial, anti-inflammatory, analgesic and antioxidant activities (Mohammadi et al., 2017 ▶).

*O. basilicum* is rich in carotenoids such as beta-carotene, which can be converted to vitamin A in the body. Beta-carotene has more antioxidant properties than vitamin A (Filip et al., 2017 ▶).

Carotenoids, especially beta-carotene, are effective in neutralizing free radicals and oxidative stress and may play a supportive role in preventing the progression of chronic diseases such as diabetes and cardiovascular disease (Ramezani et al., 2010 ▶).

Chemical study has shown that rosemarinic acid is the predominant phenolic acid in *O. basilicum* leaves and flowers (Javanmardi et al., 2002 ▶) and inhibits LDL oxidation. The inhibitory effect of rosmarinic acid on LDL oxidation may be due to its effect on the elimination of free radicals or on the deposition and chelation of copper ions, which are oxidizing agents (Azizi et al., 2004 ▶).

According to the results of the present study, it seems that the hydroalcoholic extracts of *O. persica* and *O. basilicum* can be used to decrease serum fats, inflammatory factors and PLA2 activity that increase during hypercholesterolemia. Many studies provide support for the hypothesis that marked reduction of PLA2 enzymatic activity may be an effective strategy for CVD prevention ( Burke and Dennis, 2009 ▶; Jenny, 2010 ▶; Garcia HM, and Serruys PW, 2009 ▶). Also, according to the obtained results, the cause of changes in the amount of factors can be attributed to the presence of specific flavonoid and antioxidant compounds in the mentioned plants. In the end, it may not be possible to say that the use of extracts of these plants can prevent heart disease, but they will certainly be effective in reducing their incidence.

## Conflicts of interest

The authors have declared that there is no conflict of interest.
